# Guidewire Ablation within the Coronary Venous System for Epicardial or Intramural Ventricular Arrhythmia: A Preclinical Study of Biophysical Characterization

**DOI:** 10.1155/2024/4412758

**Published:** 2024-01-04

**Authors:** Fengqi Xuan, Zhongyin Zuo, Jie Zhang, Shibei Zhang, Zichen Liu, Yunfan Meng, Kuo Sun, Yaling Han, Ming Liang, Zulu Wang

**Affiliations:** ^1^Department of Cardiology, General Hospital of Northern Theater Command, Shenyang 110016, China; ^2^Department of Cardiology, Tianjin Chest Hospital, Tianjin 300222, China

## Abstract

**Background:**

Catheter ablation failure poses a clinical challenge for epicardial or intramural ventricular arrhythmia (VA); however, guidewire ablation within the coronary venous system (CVS) may be effective and safe for targeting VAs.

**Methods:**

The ex vivo phase included four steps. In step 1, the steam pop incidence rates during guidewire ablation at power settings of 5, 10, 15, 20, and 25 W were analyzed using 10 mm- and 20 mm-tip guidewires. In step 2, guidewire ablation was performed for application durations of 10, 20, 30, 40, 50, 60, and 90 s, and the lesion size was measured. In step 3, the effects of saline infusion (0, 1, 2, 3, and 4 mL/min) on lesion dimensions and steam pop formation were examined. In step 4, an orthogonal array was constructed to obtain the optimal guidewire ablation parameters. In the *in vivo* phase, guidewire ablation within the CVS was performed in three dogs, and the lesion features in 10 days after ablation were observed.

**Results:**

In step 1, the steam pop incidence rates at 5, 10, 15, 20, and 25 W were 0%, 0%, 12.5%, 62.5%, and 100% using the 10 mm-tip guidewires and 0%, 0%, 0%, 25%, and 75% using the 20 mm-tip guidewires, respectively. In step 2, we found that the lesion areas increased with an increase in the ablation duration (the maximum lesion diameters at 30, 60, and 90 s were 4.9 ± 0.4, 7.0 ± 0.8, and 9.2 ± 0.7 mm in the 10 mm group and 3.2 ± 0.5, 4.5 ± 0.4, and 5.3 ± 0.7 mm in the 20 mm-tip group, respectively). In step 3, we observed that saline infusion was negatively correlated with ablation lesions but had a lower risk of steam pop. The optimal parameters for the 20 mm-tip guidewire ablation were 15 W, 50 s, and 2 mL/min or 20 W, 70 s, and 2 mL/min. In the *in vivo* phase, effective ablation lesions with maximum and minimum diameters of 3.2 ± 0.3 and 2.8 ± 0.5 mm, respectively, were created by the guidewires during the 10-day observation period after ablation.

**Conclusion:**

This novel radiofrequency guidewire ablation technique can feasibly create effective lesions within the CVS, which may improve the efficacy of catheter ablation for challenging epicardial or intramural VA.

## 1. Introduction

Ventricular arrhythmias (VAs) arising from the epicardial or intramural locations, especially the left ventricular summit (LVS), are particularly challenging for catheter ablation because of inadequate power delivery reaching the site of arrhythmia origin or concerns of radiofrequency (RF) collateral injuries to the coronary arteries [1, 2]. Although the efficacy of retrograde coronary venous ethanol infusion in treating failed VT has been described, it may lead to off-target myocardial injury attributable to ethanol leakage, such as AV block, ventricular damage, or pericarditis [3]. Other bailout approaches, such as bipolar ablation, needle ablation, and half-normal saline ablation, have also been reported [2, 4–6]; however, these methods are associated with a series of limitations and potential complications. Therefore, novel ablation strategies are required to treat arrhythmias.

Guidewire ablation within the coronary venous system (CVS) using RF delivery was considered as a novel ablation technique for challenging epicardial or intramural VA, and recently published clinical cases have demonstrated its feasibility by successful suppression of LVS-originated premature ventricular complexes within the CVS using a guidewire [7, 8]. However, the process of RF energy delivery by a guidewire is not yet well understood, and few experimental studies have been reported on the exploration of ablation parameters, lesion characteristics, and safety evaluation associated with guidewire ablation. This study aimed to investigate the biophysical properties of guidewire ablation within the CVS by evaluating lesion dimensions and steam pop incidence under various ablation parameters in both the 10 mm and 20 mm exposed wire-tip groups by using an ex vivo benchtop model and an *in vivo* animal model.

## 2. Methods

### 2.1. Experimental Design

This study was designed based on the following two aspects. In the ex vivo model, we examined the lesion characteristics and steam pop formation under variable power settings, ablation durations, and saline infusions to investigate the optimal ablation parameters. In step 1, we examined the steam pop incidence and lesion area under low to high power settings. In step 2, under the relative safe ablation power obtained from step 1, the relationships between the lesion dimensions and ablation durations were explored. In step 3, we assessed the effects of saline infusion on the lesion dimension and steam pop incidence under a fixed power setting and duration. In step 4, based on the results from steps 1–3, an orthogonal array was established to obtain the optimal guidewire ablation parameters. In the animal model, we further verified the ablation parameters obtained in the *in vitro* phase by analyzing lesion creation in 10 days of the survival period after guidewire ablation within the CVS.

### 2.2. Ex Vivo Experimentation

#### 2.2.1. Establishment of the Ex Vivo Ablation Model

**Figure 1:**
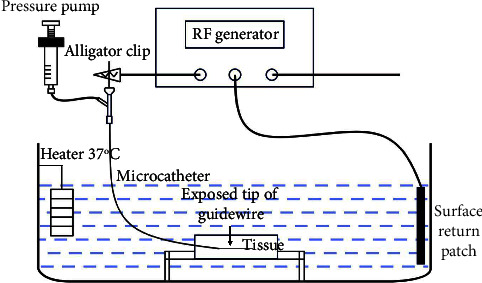
Schematic diagram of the ex vivo guidewire transvenous ablation model. A fresh porcine left ventricular myocardium with mural thickness ranging from 20 mm to 25 mm was cut into approximately 20 × 20 × 50 mm tissue slabs. Each slab was secured at the bottom of a 0.45% saline bath which was being controlled at a temperature of 37°C by a heater. The guidewire covered with a microcatheter was positioned into simulated vessels parallel to the endocardial surface, and the surface return patch was placed on the side wall. The end part of the guidewire was connected to a radiofrequency generator via an alligator clip for RF energy delivery, and to a pressure pump for saline infusion.

A distal 30 mm uninsulated guidewire (BMW; Abbott, Santa Clara, CA, USA) covered with a microcatheter (FineCross; Terumo, Tokyo, Japan) was used for ablation. The length of the wire tip could be adjusted by moving the microcatheter, and saline infusion could be performed by connecting the end of the microcatheter to the pressure pump. To better simulate the movement of the guidewire into the vessels, we established a transvenous ablation model by inserting a 6F-diameter sheath into a myocardium slab in a parallel orientation and then retreating it, and a mean caliber diameter of 1.7 ± 0.2 mm was identified under fluoroscopy by injecting a contrast agent into the simulated vessel created within the myocardium. The apparatus and electrical circuit used for the ex vivo guidewire ablation model are shown in Figure 1.

#### 2.2.2. Lesion Feature and Steam Pop Formation under Variable Ablation Parameters

We first grouped the tissue slabs based on the exposed length of the wire tip, the 10 mm and 20 mm groups, and then, guidewire ablation was performed on each group from low to high power settings: 5, 10, 15, 20, and 25 W for 60 s, with up to eight ablation applications on each power setting. The second step was to explore the lesion dimension and ablation duration. For this purpose, the lesion areas were measured at each duration time of 10, 20, 30, 40, 50, 60, and 90 s. During ablation, the guidewire was kept in a stable position relative to the target tissue, and the occurrences of steam pop, identified by an audible pop, a sudden increase in impedance, or char formation between the tissue and guidewire tip, were recorded. After ablation, the myocardial tissue was sliced into 5 mm intervals from a perpendicular orientation, and the maximum diameter (MAD) and minimum diameter (MID) of the circular cross-sectional area of the ablation lesion for at least three slices per tissue were recorded.

Saline infusion can cool the guidewire surface and ensure effective RF energy delivery. However, it may also have adverse effects on lesion dimensions. Thus, the third step aimed to assess the effects of saline infusion at various irrigation speeds on lesion size and steam pop formation. In this setting, guidewire ablation was performed at saline infusion speeds of 0, 1, 2, 3, and 4 mL/min, and the relationship between the lesion dimensions and variable infusion speeds was described.

To further optimize the guidewire ablation parameters, an orthogonal array (L9(3^4^)) was constructed to evaluate the effects of ablation power, duration, and infusion speed. The factors and experimental data for the 20 mm-tip guidewire are displayed in Table 1.

### 2.3. *In Vivo* Experimentation

#### 2.3.1. Animal Preparation and Ablation Protocol

The animal study was approved by the Institutional Animal Care and Use Committee of the General Hospital of the Northern Theater Command. Three beagle dogs weighing 20–30 kg underwent general anesthesia with intravenous propofol and were put on mechanical ventilation. Heparin was administered, and continuous ECG monitoring was performed during the entire procedure.

After vascular sheaths were introduced percutaneously into the right femoral vein, coronary venography was performed in two planes: the distal part of the great cardiac vein (GCV) and its branches, which are clearly visualized on venography, were identified as ablation targets, and two to three lesions were made on each animal with the distance between the two sites being >10 mm. A 0.014-inch-diameter guidewire covered with a microcatheter was advanced into the coronary sinus by using a guiding catheter (XBRCA 6F Cordis; Biosense Webster) under fluoroscopic guidance to reach the ablation targets. We advanced the guidewire and withdrew the microcatheter from the selected vein, leaving 20 mm of the wire tip exposed for ablation. Before ablation, selective coronary arteriography was performed to avoid the potential risk of arterial injury. Ablation sites near the coronary artery (<5 mm) were excluded. During the ablation procedure, both baseline impedance and the decrease in impedance were monitored, and the values of the power setting, ablation duration, and irrigation flow rate were based on the *in vitro* experiments described earlier. In this animal model, the steam pop was identified by an audible sound during ablation, tactile feedback, or sudden changes in impedance. Char formation was detected by careful examination of the wire tip after completing the ablation procedure. After the ablation procedure, the animals recovered and survived for 10 days.

#### 2.3.2. Lesion Analysis in Gross and Microscopic Histology

Each animal was euthanized on the 10th day after ablation, the arrested heart was excised, and careful inspection was performed for the presence of epicardial ablation lesions. We dissected the heart to identify the ablation lesions by comparing their locations on fluoroscopic images taken during the ablation procedure. Each ablation lesion was sliced at 5 mm intervals in a perpendicular orientation, and the cross-sectional area of the lesions was analyzed by measuring both the MAD and MID. Furthermore, the tissue collected at necropsy was fixed in 10% neutral-buffered formalin for at least 48 h. Then, the tissue was processed and sectioned into 10 × 10 × 5 mm pieces for dehydration, paraffin embedding, and hematoxylin-eosin and Masson's trichrome staining. The histological features of the ablation lesions were evaluated by a pathologist blinded to the intervention.

### 2.4. Statistical Analysis

Descriptive statistics were expressed as mean ± SDs for continuous variables and as frequencies or percentages for categorical variables. Guidewire ablation between variable power settings and ablation durations was compared using one-way ANOVA for normally distributed data and the Kruskal–Wallis test for others. The 10 mm and 20 mm groups were compared by using the independent sample *t*-test. Statistical significance was set at *p* < 0.05. The correlation coefficient (R) between saline infusion and ablation lesion dimension was evaluated using the Pearson correlation coefficient. A correlation coefficient of ≥0.75 was considered strong, whereas that of ≤0.5 was considered weak. Ninety-five percent of the correlations were based on the Fisher transformation. All statistical analyses were performed using SPSS version 27.0 software (SPSS Inc., Chicago, IL, USA).

## 3. Results

### 3.1. *In Vitro* Ablation

#### 3.1.1. Effect of Ablation Power on Steam Pop Formation

**Figure 2:**
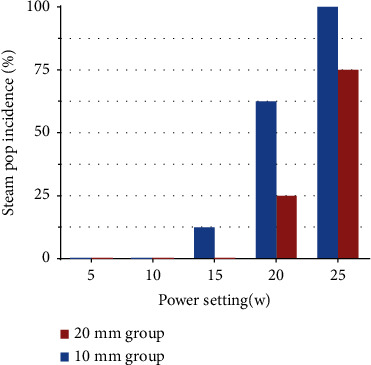
Steam pop incidence at variable power setting. Bar graphs comparing steam pop incidence at power settings of 5, 10, 15, 20, and 25 w and 10 mm versus 20 mm group comparisons were made within the same power setting group.

This study included 40 fresh swine hearts for the *in vitro* evaluation. In step 1, 80 ablation lesions were created (10 mm group, *n* = 40; 20 mm group, *n* = 40).  Table 2 shows the ablation characteristics and lesion dimensions created at variable power settings. We found that the occurrence of steam pops significantly increased with an increase in the ablation power; in the 10 mm group, fewer steam pops occurred at 5 (0/8, 0%), 10 (0/8, 0%), and 15 (1/8, 12.5%) W, whereas at higher power settings of 20 and 25 W, the steam pop incidence rates were 62.5% and 100% at 60 s, respectively. Thus, we deduced that a power setting of 10 or 15 W was safe for 10 mm exposed wire ablation. Compared with the 10 mm group, the 20 mm exposed wire group showed a lower rate of steam pop at similar ablation powers; ablation data from the 20 mm group showed steam pop incidence rates of 0%, 0%, 0%, 25%, and 75% at 5, 10, 15, 20, and 25 W, respectively, which indicated a safe power setting of 15 W or 20 W for the 20 mm group (Figure 2). Additionally, as shown in Table 2, the lesion areas increased with an increase in the ablation power (MAD: 1.9 ± 0.3, 3.5 ± 0.6, 4.4 ± 0.5, and 4.9 ± 0.6 mm at 5, 10, 15, and 20 W, respectively, in the 20 mm group (*p* < 0.01); MID: 1.5 ± 0.3, 3.1 ± 0.6, 3.8 ± 0.5, and 4.3 ± 0.6 mm (*p* < 0.01), respectively), and ablation with the 10 mm exposed wire resulted in larger lesions than ablation with the 20-mm exposed wire at a similar power setting and ablation duration (MAD: 2.7 ± 0.4 vs. 1.9 ± 0.3, 4.7 ± 0.8 vs. 3.5 ± 0.6, and 6.6 ± 0.5 vs. 4.4 ± 0.5 mm at power settings of 5, 10, and 15 W, respectively (*p* ≤ 0.05 for all comparisons); MID: 2.0 ± 0.3 vs. 1.5 ± 0.3 mm at 5 W (*p*=0.07), 3.9 ± 0.2 vs. 3.1 ± 0.6 mm at 10 W (*p*=0.02), and 5.9 ± 0.4 vs. 3.8 ± 0.5 mm at 15 W (*p* < 0.01)).

#### 3.1.2. Effect of the Ablation Duration on the Lesion Dimension

**Figure 3:**
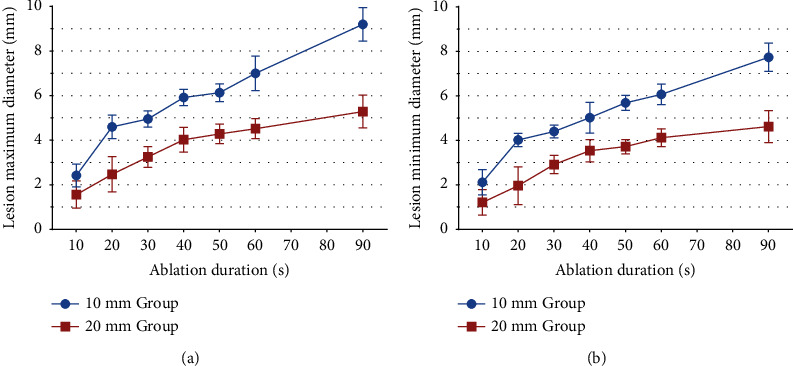
Lesion dimension along with ablation duration. (a) Maximum diameter of ablation lesion at 10, 20, 30, 40, 50, 60 and 90 seconds. (b) Minimum diameter of ablation lesion at 10, 20, 30, 40, 50, 60 and 90 seconds. Note the larger lesions created by 10 mm-tip guidewire at each ablation duration.

**Figure 4:**
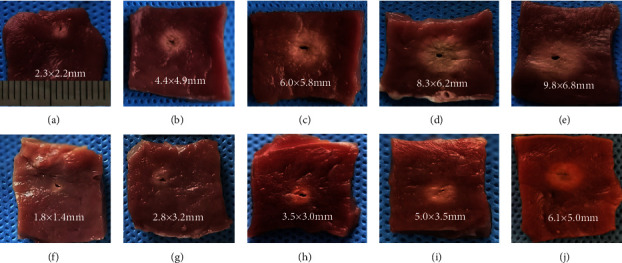
Example of the gross lesion in ex vivo ablation of 10 mm-tip guidewires at a power setting of 15 w with infusion speed of 2 ml/min for 10, 30, 50, 60, and 90 seconds, respectively (a–e). Ablation of 20 mm-tip guidewire at the similar ablation duration of 10, 30, 50, 60, and 90 seconds (f–j).

In step 2, we compared the lesion dimensions by guidewire ablation at variable application durations and found that the ablation lesion enlarged with an increase in the ablation time.  Table 3 and Figure 3 show the lesion dimensions at a power setting of 15 W for 10, 20, 30, 40, 50, 60, and 90 s (*n* = 6 for each time point) in the 10 mm and 20 mm groups. An example of a gross lesion image for ablation durations of 10, 30, 50, 60, and 90 s is shown in Figure 4. In the 20 mm-tip group, ablation lesions with an MID of >4 mm were produced when the ablation duration was up to 60 s. Additionally, the baseline impedance range and decrease in impedance at variable ablation durations in both the 10 mm and 20 mm groups were recorded in the ex vivo model. As shown in Table 3, the baseline impedance in the 20 mm group ranged from 130 to 150 Ω, which was lower than the 10 mm group (170–210 Ω, *p* < 0.01 for all comparisons at ablation durations of 10, 20, 30, 40, 50, 60, and 90 s). The magnitudes of the decrease in impedance that was calculated by subtracting the terminal impedance value from the baseline impedance value were 57.0 ± 3.7 versus 28.3 ± 5.0, 57.0 ± 3.7 versus 28.3 ± 5.0, and 57.0 ± 3.7 versus 28.3 ± 5.0 Ω at ablation durations of 30, 60, and 90 s in the 10 mm and 20 mm groups, respectively. During ablation in step 2, steam pop occurred only once at an ablation duration of 90 s in the 10 mm-tip group, and no char formation was detected surrounding the guidewire.

#### 3.1.3. Effect of Saline Infusion on the Lesion Dimension and Steam Pop

**Figure 5:**
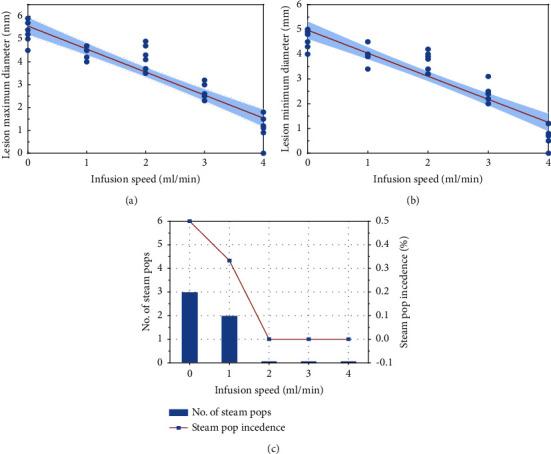
The relationship between saline infusion and lesion size. Scatter correlation plots between saline infusion with speeds of 0 ml/min, 1 ml/min, 2 ml/min, 3 ml/min, and 4 ml/min, and ablation lesion's (a) maximum diameter and (b) minimum diameter. (c) Numbers of steam pop and steam pop incidence at each saline infusion speed.

In step 3, we found that the saline infusion resulted in a strong negative effect on the lesion area but a lower rate of steam pop. At saline irrigation speeds of 0, 1, 2, 3, and 4 mL/min, the MADs of the lesions were 5.3 ± 0.5, 4.4 ± 0.3, 4.2 ± 0.5, 2.8 ± 0.4, and 1.1 ± 0.6 mm (R: 0.93; CI: 5.17–5.96; Figure 5(a)), and the MIDs were 4.6 ± 0.4, 4.1 ± 0.4, 3.8 ± 0.4, 2.4 ± 0.4, and 0.7 ± 0.5 mm (R: 0.92; CI: 4.60–5.34; Figure 5(b)), respectively; however, the numbers of steam pops were 3/6, 2/6, 0/6, 0/6, and 0/6, respectively. Notably, an infusion speed of 2 mL/min allowed the maximum lesion size with reduced steam pop incidence.

#### 3.1.4. Optimizing the Guidewire Ablation Parameters

The experimental data obtained from the orthogonal design are listed in Table 4. The optimal parameters for the 20 mm-tip guidewire ablation, which produced the maximum lesion size but lower rate of steam pop, were as follows: 15 W, 2 mL/min, and 50 s (MAD: 4.45; MID: 3.05; number of steam pops: 0/8) and 20 W, 2 mL/min, and 70 s (MAD: 5.02; MID: 4.39; number of steam pops: 1/8). According to the largest donating rule, accumulated based on the MAD of the ablation lesion, the factor with the largest range value (Kmax-Kmin) has the greatest effect on lesion formation after ablation. Table 4 shows that the rank order of the three factors was *R*_power_ > *R*_infusion_ > *R*_duration_. This result indicated that the ablation power had the greatest impact on the lesion area, followed by saline infusion. Additionally, the ANOVA results indicated that ablation power, saline infusion, and ablation duration all were significant factors (Table 5).

### 3.2. *In Vivo* Experiment

A total of nine ablation applications were successfully performed within the coronary veins by the guidewire with the 20 mm-exposed tip in three animals, using the optimal ablation parameters obtained from an *in vitro* experiment (power setting of 15 W for 60 s with the saline infusion speed of 2 mL/min). One application was discarded because of the occurrence of steam pop, which was identified as a sharp increase in impedance. Neither VA nor ischemic ST-T changes were detected on ECG during the entire procedure. After ablation, the wire tip was carefully examined, and no char formation was detected.  Table 6 shows the detailed biophysical ablation data and lesion features *in vivo*.

**Figure 6:**
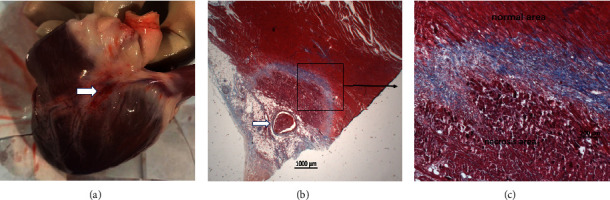
An example of a guidewire ablation lesion. (a) Gross lesion located at the distal segment of GCV 10 days postablation. (b) Histology examination showed a well-demarcated lesion around the ablation vessel via Masson's trichrome staining (MT, 1×). (c) Junction of ablation lesion and normal myocardium (note: necrosis tissue was partly replaced by fibrosis (MT, 5×)).

Gross examination revealed clear lesions in six of the eight applications, which were located at the distal segment of the GCV (four lesions) and branches of the GCV (two lesions); two lesions were excluded because no visible ablated myocardium was found. Similar to the observation in the ex vivo benchtop model, effective and consecutive lesions could be created by the guidewire within the CVS at 10 days after ablation (MAD: 3.2 ± 0.3 mm; MID: 2.8 ± 0.5 mm; *n* = 6). However, the lesions created *in vivo* were not as wide as those created in the ex vivo model at similar ablation parameters (MAD: 3.2 ± 0.3 vs. 4.2 ± 0.5 mm (*p*=0.02); MID: 2.8 ± 0.5 vs. 3.8 ± 0.4 mm (*p*=0.03)). As depicted in Figure 6(a), some gross lesions at 10 days after ablation showed obvious epicardial hyperemia, which may be associated with an acute inflammatory reaction; however, no pericardial effusion was detected. Histological examination showed myocardium necrosis around the target veins in all (6/6) tissue sections. In the representative examples shown in Figures 6(b) and 6(c), the lesions were well demarcated from the surrounding normal tissue, and necrosis tissues were partly replaced by fibrosis.

## 4. Discussion

In this study, we examined the biophysical features of guidewire ablation with respect to the lesion dimensions and safety using variable ablation parameters in both ex vivo and *in vivo* experiments. This investigation has major findings. First, the results from guidewire ablation in the ex vivo model showed that the steam pop incidence increased with an increase in the power setting, and ablation with the 10 mm-tip guidewire resulted in a higher rate of steam pop than that in the 20 mm-tip group at a similar power setting. Second, the lesion areas increased with an increase in the ablation duration, and ablation using the 10 mm-tip guidewires produced larger lesions than that with the 20 mm-tip guidewires at a similar ablation time. This was because of the higher current delivery on the relatively smaller surface area of the 10 mm exposed tip than on that of the 20 mm exposed tip. Moreover, saline infusion had a strong negative correlation with lesion dimensions but a lower rate of steam pop. Based on the optimization study, the optimal parameters for the 20 mm-tip guidewire ablation were the following: 15 W, 2 mL/min, and 50 s or 20 W, 2 mL/min, and 70 s. Lastly, in the animal model, guidewire ablation within the CVS created effective and safe lesions under the optimal ablation data obtained from the ex vivo model without VA or ischemic ST-T changes on ECG monitoring.

Guidewire ablation within the CVS is a novel technique for the treatment of failed VA originating from the epicardial or intramural locations. As a commonly used tool for a coronary intervention, the guidewire was first described for intravascular mapping to guide ablation in electrophysiology [3, 9, 10], which offers a novel mapping strategy for targeting the epicardially originating VA. The clinical application of guidewire ablation was first described by Romero et al. [11]. They advanced a stiff stingray guidewire used for chronic total occlusions of the coronary artery into the interventricular septal myocardium through the first septal perforator artery for both mapping and ablation in the treatment of intramural LVS VA. Subsequently, a distal 15 mm uninsulated vision guidewire with a soft tip was reported by Efremidis for both unipolar mapping and ablation at the distal segment of the GCV [8]. This was the first description of guidewire ablation within the CVS for terminating the LVS VA. Recently, we published another case report on guidewire ablation within the distal GCV for the treatment of epicardial VA [7]. Unlike the single guidewire utilized in the previous study, a distal 30-mm uninsulated guidewire covered with a microcatheter was used in the current study. The potential advantage of this novel technique is that the length of the exposed wire tip can be adjusted by moving the microcatheter because a shorter exposed tip contributes to recording a more accurate unipolar signal, whereas a longer exposed tip can be suitable for ensuring that the area of interest is completely covered. Another advantage is the ability to perform saline infusion by using a microcatheter, which may be particularly valuable because it reduces wire surface temperature and intravenous impedance.

However, the biophysical ablation properties of guidewires have not yet been reported. In this preclinical study, we examined the efficacy of this novel technology by using guidewires covered with a microcatheter for RF ablation. The results of our ex vivo experiments suggest that contiguous and effective lesions can be created by using a guidewire. We first compared variable ablation powers with lesion formations and found that the lesion size increased with an increase in the ablation power; however, a higher power setting was associated with steam pop incidence. The ablation duration was also related with the lesion dimension and tissue heating, and effective lesions (MAD of 5.9 ± 0.4 mm and MID of 5.0 ± 0.7 mm by the 10 mm-tip guidewire at 40 s and 4.5 ± 0.4 and 4.1 ± 0.4 mm by the 20 mm-tip guidewire at 60 s) were achieved with a power setting of 15 W and infusion speed of 2 mL/min, which was adequate for clinical ventricular ablation with epicardial origins. To evaluate the optimal combination of ablation parameters, an orthogonal test design was used, and the optimal parameters of 15 W, 2 mL/min, and 50 s or 20 W, 2 mL/min, and 70 s were identified; the power setting was viewed as the largest donating factor on the lesion dimension. We further assessed the value of the ablation parameters (15 W, 2 mL/min, and 60 s) in predicting the lesion dimension created by guidewire RF ablation in the animal model, since most of our clinical physicians have a common practice of choosing ablation durations of 30 s, 45 s, or 60 s. During the 10-day observation period after ablation, the effective and demarcated lesion was created at a gross level without safety issues, which suggests that the titration of the RF dose we explored ex vivo is satisfactory. However, the lesions created *in vivo* were not as wide as those in the isolated myocardium at similar ablation parameters (MAD: 3.2 ± 0.3 vs. 4.2 ± 0.5 mm (*p*=0.02); MID: 2.8 ± 0.5 vs. 3.8 ± 0.4 mm (*p*=0.03)). In addition, two of the eight guidewire ablation applications *in vivo* were abandoned because no visible lesion was found. This is because unlike isolated myocardium ablation in which the relationship between power, duration, and lesion formation is fairly straightforward, guidewire ablation *in vivo* involves complex and uncontrollable parameters, such as the presence of epicardial fat, the caliber of the target vessels, and contact area of the wire and tissue, which also influence the lesion dimension caused by guidewire ablation.

RF ablation with a higher power setting, longer duration, or lower infusion speed, which is required to improve lesion dimensions, could inevitably increase the risk of tissue overheating and steam pop formation. Therefore, the safety and efficacy ranges of guidewire ablation should be studied. For the 20 mm-tip guidewire, a power setting of 15 or 20 W and saline infusion of 2 mL/min were considered safe parameters by the *in vitro* experiment; subsequently, these parameters were verified in the animal study, and a satisfactory steam pop incidence (1/9) was detected. In addition, we observed that the steam pop incidence under optimal power setting and saline infusion was relatively lower even at a longer ablation duration (0/6 in the 20 mm group at 15 W, 2 mL/min, and 90 s), which indicated that it is safer to increase the ablation duration than to increase the power setting for larger lesions. In addition to evaluating the optimal ablation parameters, the length of the exposed tip was also noteworthy because it correlated with the current output and distribution. Wire tips with smaller surface areas share the higher current output, which may produce larger lesion areas but also easily result in tissue overheating. Therefore, audible pop or char formation at the wire tip should be noted, and a lower ablation power may be suitable for shortening the exposed tip of the guidewire during ablation. Saline infusion from the microcatheter in our study contributed to preventing char formation at the wire tip and increasing the safety of this approach. However, saline infusion can also produce negative effects on the lesion size. Thus, we assessed the effects of various saline infusion speeds on the lesion dimensions and steam pop incidence and found an optimal infusion speed of 2 mL/min, which balances the efficacy and safety of guidewire ablation.

The limitation of guidewire ablation lies in the fact that the surface temperature of the wire tip cannot be monitored; therefore, there may be high risks of overheating or char formation at the wire tip, which suggests a deeper tissue temperature exceeding 100°C. Saline infusion plays a role in preventing char formation during guidewire ablation; however, the efficacy of saline irrigation *in vivo* could be influenced by many uncontrollable factors. Therefore, in a clinical setting, char formation may be a limitation of this technique, titration of RF energy delivery should be used, and examination of the wire tip in time is required.

## 5. Conclusion

Data from both *in vitro* and *in vivo* studies suggest that effective lesions could be safely created by guidewire ablation within vessels. Dimensional characteristics of guidewire ablation lesions are associated with ablation power, duration, saline infusion speed, and exposed tip of the guidewire. The steam pop should be noted with a higher ablation power and lower infusion speed, as well as shorter exposed wire tip.

## Figures and Tables

**Table 1:** Orthogonal design factors and levels.

Factors	Level		
	1	2	3
Power setting	10 w	15 w	20 w
Ablation duration	50 s	60 s	70 s
Infusion speed	1 ml/min	2 ml/min	3 ml/min

**Table 2:** Steam pop incidence and lesion dimension at variable power setting in an ex vivo model.

	10 mm group ablation time: 60 s; saline infusion: 2 ml/min						20 mm group ablation time: 60 s; saline infusion: 2 ml/min					
	5 w (*n* = 8)	10 w	15 w	20 w	25 w	*p*value	5 w (*n* = 8)	10 w	15 w	20 w	25 w	*p*value
Baseline impedance (Ω)^*∗*^	200.3 ± 10.9	197.0 ± 13.9	195.0 ± 10.4	194.0 ± 10.2	195.0 ± 12.0	0.849	146.6 ± 5.2	135.9 ± 11.1	141.4 ± 8.6	145.4 ± 8.8	141.9 ± 9.9	0.159
No. of steam pops	0/8	0/8	1/8	5/8	8/8	—	0/8	0/8	0/8	2/8	6/8	—
Time to steam pop (second)	—	—	40	27.5 ± 10.4	8.2 ± 3.2	—	—	—	—	49.0 ± 5.7	25.7 ± 7.2	—
Lesion's maximum diameter (mm)	2.7 ± 0.4	4.6 ± 0.8	6.6 ± 0.5	—	—	<0.01	1.9 ± 0.3	3.5 ± 0.6	4.4 ± 0.5	4.9 ± 0.6	—	<0.01
Lesion's minimum diameter (mm)	2.0 ± 0.3	3.9 ± 0.2	5.8 ± 0.4	—	—	<0.01	1.5 ± 0.3	3.1 ± 0.6	3.8 ± 0.5	4.3 ± 0.6	—	<0.01

^*∗*^Ω indicates ohm.

**Table 3:** Ablation characteristics and lesion dimension at variable ablation duration in an ex vivo model.

	10 mm group	20 mm group	*p*value	10 mm group	20 mm group	*p*value	10 mm group	20 mm group	*p*value	10 mm group	20 mm group	*p*value	10 mm group	20 mm group	*p*value	10 mm group	20 mm group	*p*value	10 mm group	20 mm group	*p*value
	10 s			20 s			30 s			40 s			50 s			60 s			90 s		
Baseline impedance (Ω)^*∗*^	195.8 ± 18.2	138.0 ± 6.9	<0.01	194.0 ± 15.1	141.3 ± 8.1	<0.01	190.0 ± 12.9	142.8 ± 8.6	<0.01	193.8 ± 17.2	145.0 ± 10.5	<0.01	192.5 ± 16.9	144.0 ± 11.0	<0.01	194.2 ± 14.9	138 ± 5.6	<0.01	194.3 ± 11.7	142.2 ± 10.4	<0.01
Impedance drop (Ω)^*∗*^	45.2 ± 4.9	22.0 ± 4.1	<0.01	52.7 ± 4.0	26.2 ± 3.9	<0.01	57.0 ± 3.7	28.3 ± 5.0	<0.01	59.2 ± 4.3	31.2 ± 3.3	<0.01	62.2 ± 3.9	30.8 ± 3.4	<0.01	64.0 ± 4.4	31.3 ± 4.3	<0.01	65.8 ± 3.3	33.8 ± 4.3	<0.01
Steam pop incidence (%)	0	0	—	0	0	—	0	0	—	0	0	—	0	0	—	0	0	—	17	0	—
Lesion's maximum diameter (mm)	2.4 ± 0.5	1.6 ± 0.6	0.025	4.6 ± 0.5	2.5 ± 0.8	<0.01	4.9 ± 0.4	3.2 ± 0.5	<0.01	5.9 ± 0.4	4.0 ± 0.5	<0.01	6.1 ± 0.4	4.3 ± 0.4	<0.01	7.0 ± 0.8	4.5 ± 0.4	<0.01	9.2 ± 0.7	5.3 ± 0.7	<0.01
Lesion's minimum diameter (mm)	2.1 ± 0.6	1.2 ± 0.6	0.02	4.0 ± 0.3	1.9 ± 0.8	<0.01	4.4 ± 0.3	2.9 ± 0.4	<0.01	5.0 ± 0.7	3.5 ± 0.5	0.02	5.7 ± 0.3	3.7 ± 0.3	<0.01	6.0 ± 0.5	4.1 ± 0.4	<0.01	7.7 ± 0.6	4.6 ± 0.7	<0.01

^*∗*^Ω indicates ohm.

**Table 4:** Orthogonal design and experimental results.

No.	Power (w)	Duration (s)	Infusion speed (ml/min)	Maximum diameter (mm)	Steam pop incidence
1	10	50	1	3.60	0/8
2	10	60	2	3.20	0/8
3	10	70	3	2.10	0/8
4	15	50	2	4.22	0/8
5	15	60	3	3.10	0/8
6	15	70	1	5.25	3/8
7	20	50	3	3.41	0/8
8	20	60	1	5.40	4/8
9	20	70	2	5.13	1/8
K1	2.96	3.74	4.75	—	
K2	4.19	3.89	4.17		
K3	4.65	4.16	2.87		
R	1.69	0.42	1.88		

**Table 5:** Analysis of variance of three guidewire ablation parameters.

Source	Sum of square	Degree of freedom	F-ratio	P value
Power setting	4.59	2	1315.1	0.001
Ablation duration	0.26	2	75.6	0.013
Infusion speed	5.58	2	1598.6	0.001
Error	0.003	2	—	

**Table 6:** Ablation parameters of guidewire ablation in the animal model.

Power setting	15 w	No. of guidewire ablation	9
Ablation duration	60 seconds	No. of steam pop	1
Exposed length of the wire tip	20 mm	Time to steam pop	35 seconds
Saline infusion speed	2 ml/min	Lesion maximum diameter	3.2 ± 0.3 mm
Baseline impedance	170 ± 13.5 Ω	Lesion minimum diameter	2.8 ± 0.5 mm
Impedance drop	11.8 ± 3.7 Ω	Survival time	10 d

## Data Availability

The statistical and image data used to support the finding of this study are included within the article.
